# Supplementary material for: GATA2 mitotic bookmarking is required for definitive haematopoiesis
Source: Nat Commun. 2023 Aug 14;14:4645. doi: 10.1038/s41467-023-40391-x (PMC10425459; doi:10.1038/s41467-023-40391-x)

GATA2 mitotic bookmarking is required for definitive haematopoiesis

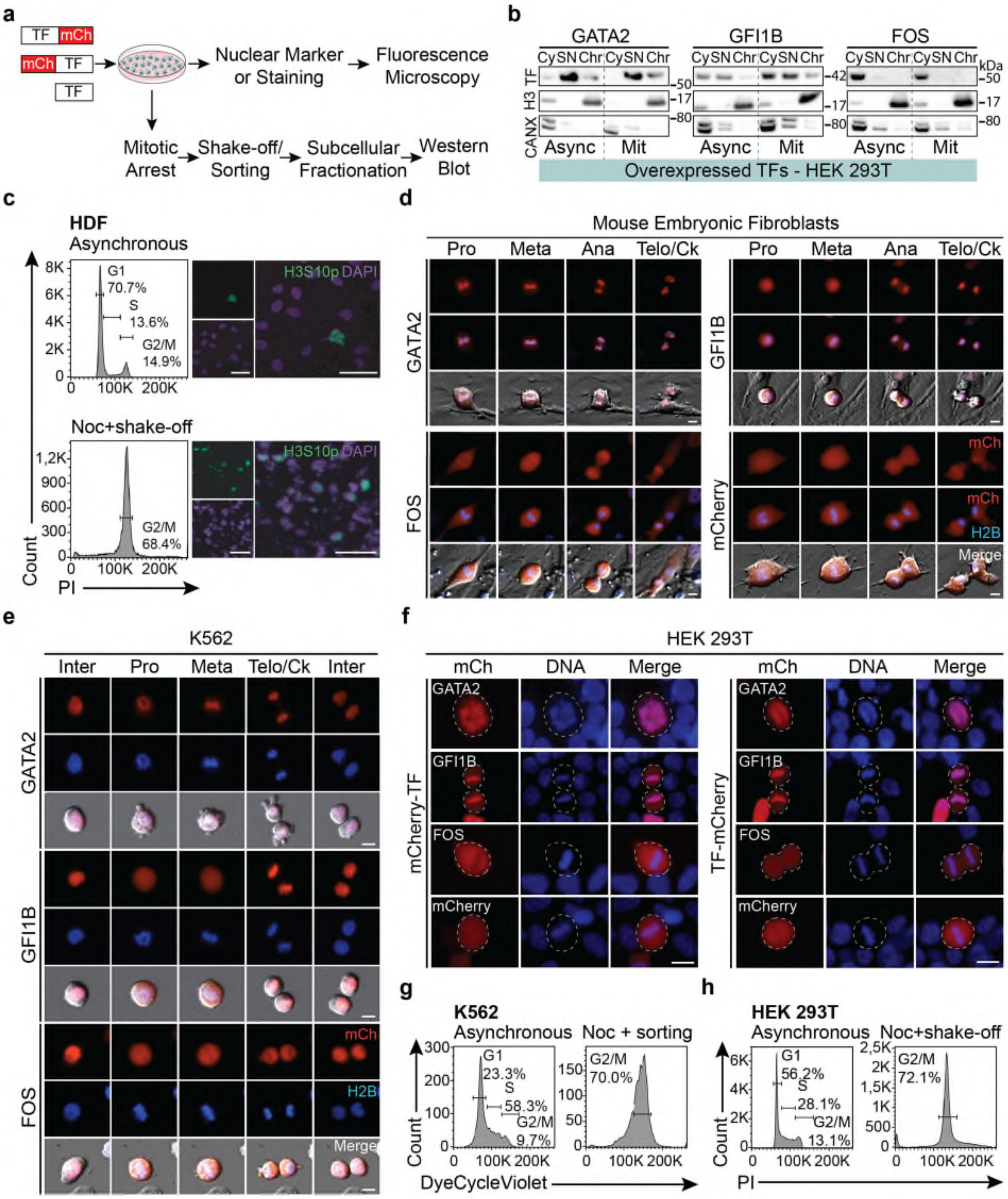

Supplementary Fig. 1. GATA2, GFI1B and FOS chromatin retention in mitotic cells. a, Strategy to assess mitotic chromatin retention. Individual transcription factors (TFs) were fused to mCherry (mCh) fluorescent protein upstream or downstream TF sequence and mouse embryonic fibroblasts, human dermal fibroblasts (HDFs), K562 or HEK 293T cells analysed by live-cell

imaging. In parallel, HDFs, K562 or HEK 293T cells were arrested in pro-metaphase with nocodazole followed by subcellular protein fractionation and protein levels were detected by western blot. **b,** TF expression in cytoplasmic (Cy), soluble nucleus (SN) and chromatin-bound (Chr) protein fractions of asynchronous (Async) and mitotic (Mit) HEK 293T cells, after overexpression of the indicated TF. Calnexin (CANX) and histone 3 (H3) represent loading controls. Western blots were performed once. kDA, kilodaltons. **c,** Flow cytometry cell cycle analysis (left) and immunofluorescence (right) for the mitotic marker histone H3 phosphorylated at serine 10 (H3S10p) in asynchronous and mitotic HDFs after nocodazole (Noc) treatment and mitotic shake-off. Nuclei were stained with DAPI. Scale bar = 100 µm. Experiment was performed once. **d**, Live-cell images of mouse embryonic fibroblasts overexpressing mCh-TF fusion proteins during mitosis (Pro – prophase, Meta – metaphase, Telo/Ck – Telophase/Cytokinesis). DNA content is represented by histone 2B (H2B)-mTurquoise signal. Scale bar = 10 µm. Mitotic events: n(GATA2)=31, n(FOS)=36, n(GFI1B)=31, n(mCherry)=19. **e**, Live-cell images of K562 cells overexpressing mCh-GATA2 or mCh-GFI1B fusions during interphase (Inter) and mitosis. Mitotic events: n(GATA2)=28, n(FOS)=35, n(GFI1B)=74. Scale bar = 10 µm. **f**, Live-cell images of HEK 293T cells overexpressing mCherry fused to the N-terminal (mCherry-TF) or C-terminal (TF-mCherry) of the 3 TFs during mitosis. Hoechst marks DNA. Scale bar = 10 µm. **g**, Flow cytometry cell cycle analysis of asynchronous and nocodazole (Noc) treated K562 cells with the permeable DNA stain Vybrant DyeCycle Violet. For western blot quantifications, live mitotic cells were FACS sorted using the indicated G2/M gate. **h**, Cell cycle analysis of asynchronous and nocodazole (Noc) arrested HEK 293T cells after mitotic shake-off. Experiment was performed once. PI – propidium iodide.

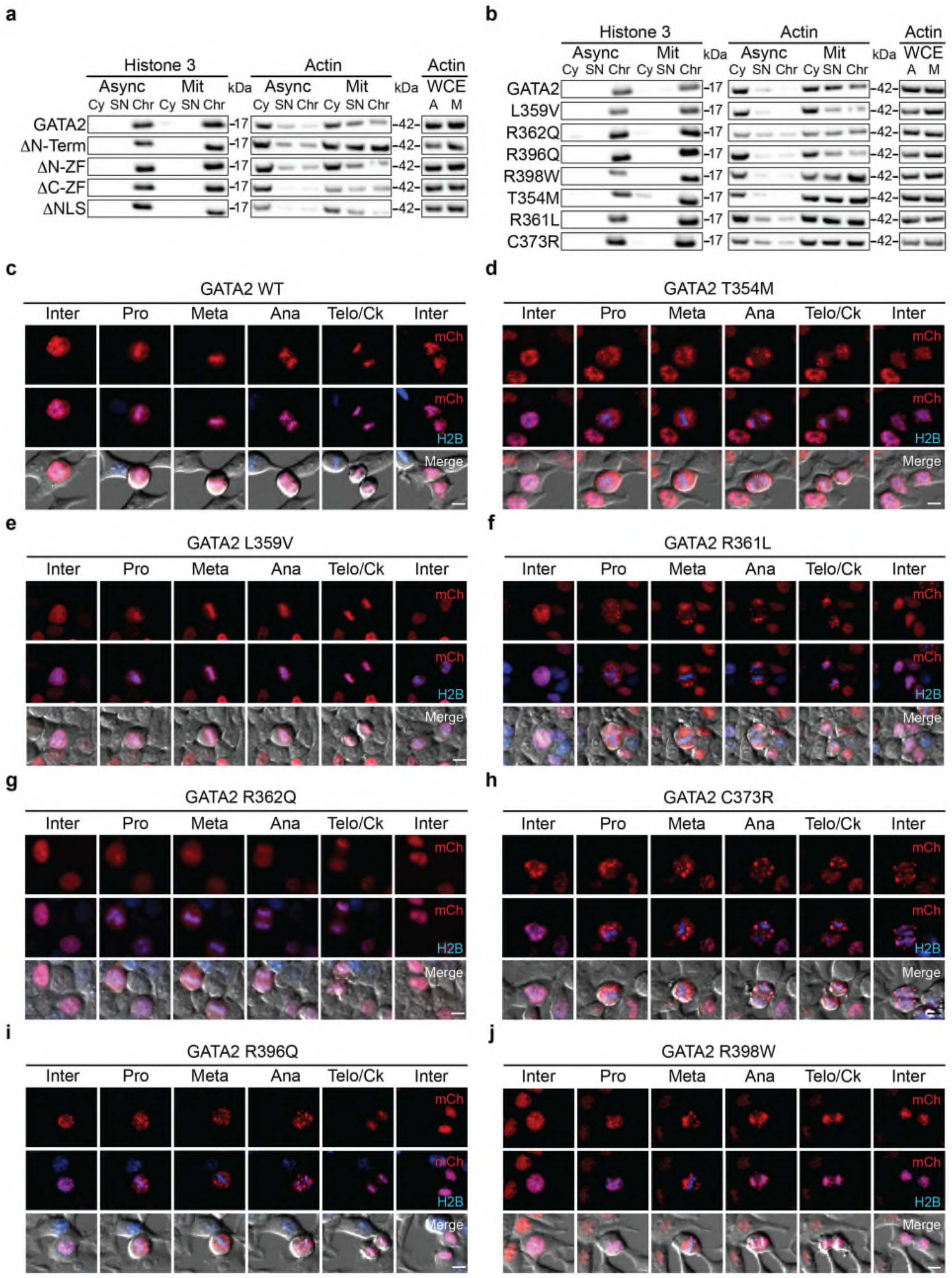

Supplementary Fig. 2. Chromatin retention of GATA2 is reduced by mutations in the C-terminal zinc finger (C-ZF). a, **b**, Western blot analysis of actin and histone 3 loading controls

for mCherry-GATA2 deletion constructs (**a**) and mutant proteins (**b**) in whole-cell extracts (WCE) and protein fractionations of asynchronous (A, Async) and mitotic (M, Mit) HEK 293T cells. Bands were acquired using the same exposure times for asynchronous and mitotic cells, depending on the antibody and protein isolation method. Western blots were repeated at least three times. Cy, cytoplasmic protein fraction. SN, soluble nucleus protein fraction. Chr, chromatin-bound protein fraction. kDA, kilodaltons. **c-j**, Live-cell images of HEK 293T cells overexpressing mCherry (mCh)-GATA2 (red) wild-type (WT) (**c**) and GATA2 proteins mutated in C-ZF in positions T354M (**d**), L359V (**e**), R361L (**f**), R362Q (**g**), C373R (**h**), R396Q (**i**) and R398W (**j**) in interphase (Inter) and mitosis (Pro – prophase, Meta – metaphase, Telo/Ck – Telophase/Cytokinesis). The first letter represents the wild-type amino acid, followed by the position and the replaced amino acid. DNA is marked by histone 2B (H2B)-mTurquoise (blue). Scale bars = 10 µm. Mitotic events: n(GATA2)=219, n(T354M)=724, n(L359V)=78, n(R361L)=165, n(R362Q)=18, n(C373R)=360, n(R396Q)=151, n(R398W)=147.

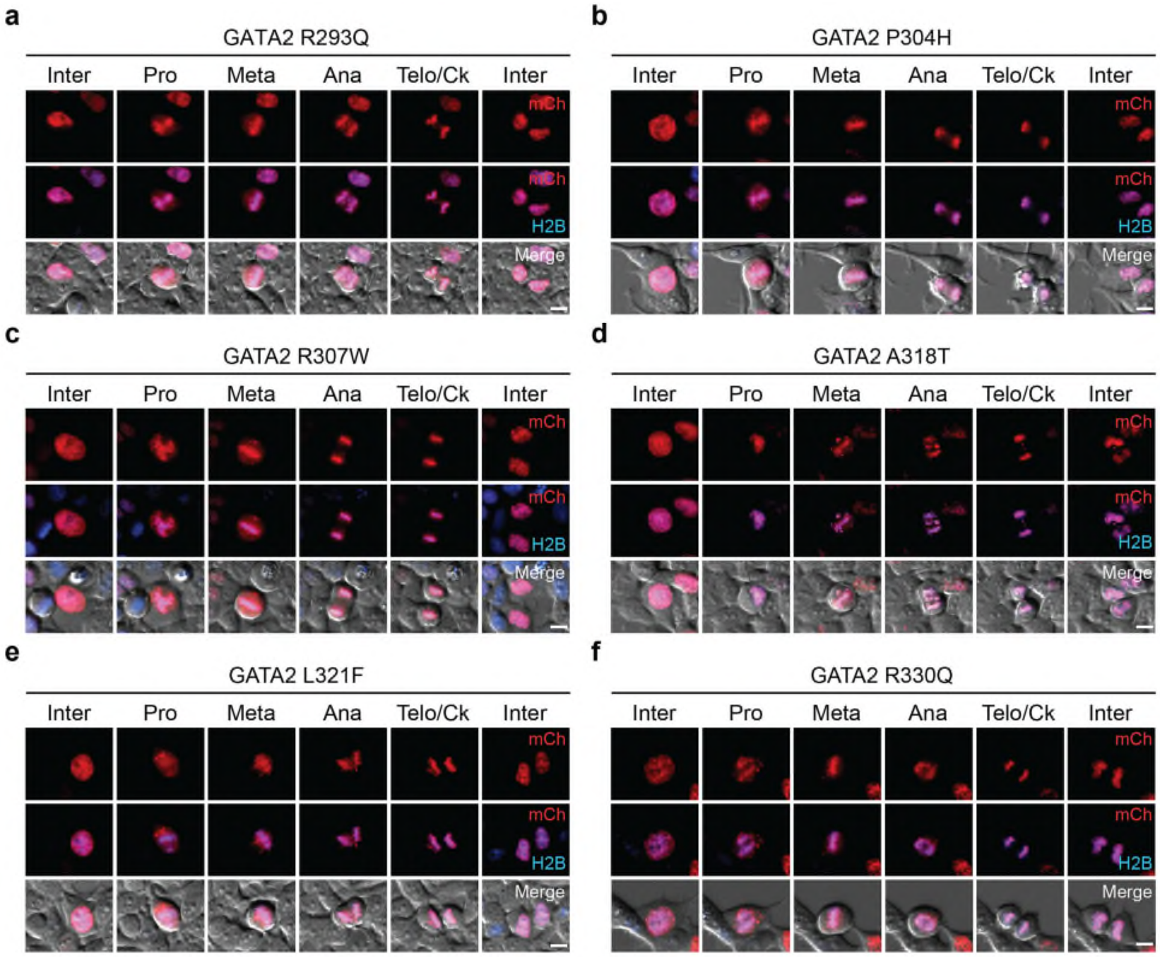

Supplementary Fig. 3. Chromatin retention of GATA2 is not impacted by mutations in the N-terminal zinc finger (N-ZF). a-f, Live-cell images of HEK 293T cells overexpressing mCherry (mCh)-GATA2 (red) proteins mutated in N-ZF in positions R293Q (**a**), P304H (**b**), R307W (**c**), A318T (**d**), L321F (**e**), R330Q (**f**) in interphase (Inter) and mitosis (Pro – prophase, Meta – metaphase, Telo/Ck – Telophase/Cytokinesis). The first letter represents the wild-type amino acid, followed by the position and the replaced amino acid. DNA is marked by histone 2B (H2B)-mTurquoise (blue). Scale bars = 10 µm. Mitotic events: n(R293Q)=73, n(P304H)=151, n(R307W)=172, n(A318T)=296, n(L321F)=290, n(R330Q)=92.

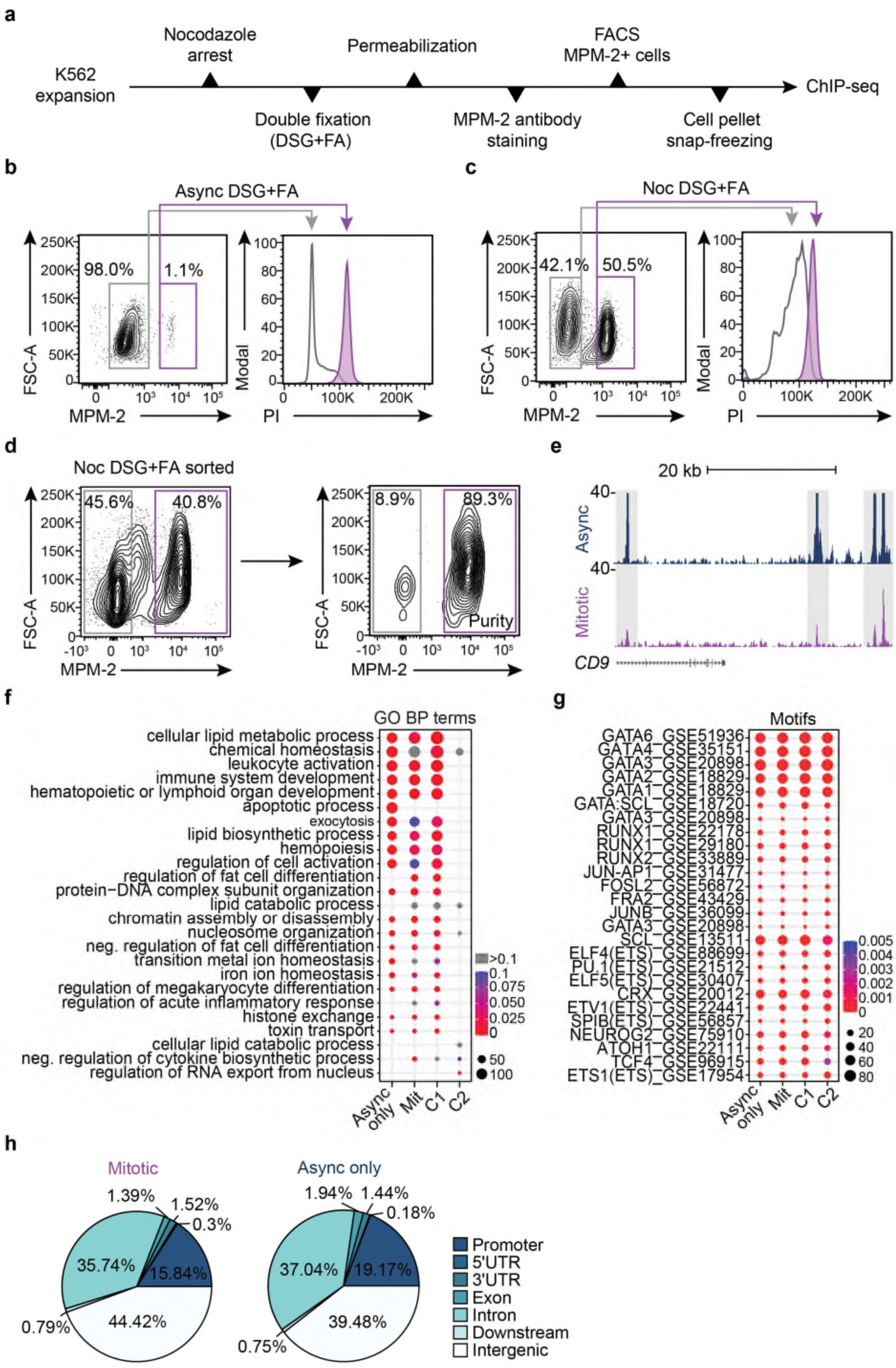
a
K562 expansion
Nocodazole arrest
Double fixation (DSG+FA)
Permeabilization
MPM-2 antibody staining
FACS MPM-2+ cells
Cell pellet snap-freezing
ChIP-seq
b
Async DSG+FA
98.0%
1.1%
FSC-A
MPM-2
Modal
PI
c
Noc DSG+FA
42.1%
50.5%
d
Noc DSG+FA sorted
45.6%
40.8%
8.9%
89.3%
Purity
e
20 kb
Async
Mitotic
CD9
f
GO BP terms
cellular lipid metabolic process
chemical homeostasis
leukocyte activation
immune system development
hematopoietic or lymphoid organ development
apoptotic process
exocytosis
lipid biosynthetic process
hemopoiesis
regulation of cell activation
regulation of fat cell differentiation
protein−DNA complex subunit organization
lipid catabolic process
chromatin assembly or disassembly
nucleosome organization
neg. regulation of fat cell differentiation
transition metal ion homeostasis
iron ion homeostasis
regulation of megakaryocyte differentiation
regulation of acute inflammatory response
histone exchange
toxin transport
cellular lipid catabolic process
neg. regulation of cytokine biosynthetic process
regulation of RNA export from nucleus
Async only
Mit
C1
C2
g
Motifs
GATA6_GSE51936
GATA4_GSE35151
GATA3_GSE20898
GATA2_GSE18829
GATA1_GSE18829
GATA:SCL_GSE18720
GATA3_GSE20898
RUNX1_GSE22178
RUNX1_GSE29180
RUNX2_GSE33889
JUN-AP1_GSE31477
FOSL2_GSE56872
FRA2_GSE43429
JUNB_GSE36099
GATA3_GSE20898
SCL_GSE13511
ELF4(ETS)_GSE88699
PU.1(ETS)_GSE21512
ELF5(ETS)_GSE30407
CRX_GSE20012
ETV1(ETS)_GSE22441
SPIB(ETS)_GSE56857
NEUROG2_GSE75910
ATOH1_GSE22111
TCF4_GSE96915
ETS1(ETS)_GSE17954
h
Mitotic
Async only
Promoter
5'UTR
3'UTR
Exon
Intron
Downstream
Intergenic

Supplementary Fig. 4. Sorting strategy for Chromatin Immunoprecipitation followed by sequencing (ChIP-seq) of mitotic K562 cells and complementary analyses. a, Outline of mitotic K562 fixation and FACS sorting steps. K562 were arrested with 0.2 μg/mL nocodazole for 12-14h and fixed with 2mM Di(N-succinimidyl) glutarate (DSG) before fixation with 1% formaldehyde (FA). After that, cells were permeabilized and stained with anti-phospho-Ser/Thr-Pro MPM-2 primary antibody and anti-mouse AF647 secondary antibody. MPM-2 antibody recognizes several phosphorylated proteins during mitosis. Cells positive for MPM-2 were FACS sorted, washed and cell pellets were snap-frozen for ChIP. **b**, Quantification of interphasic and mitotic K562 cells after double fixation. Mitotic cells were identified in the 4N peak according to propidium iodide (PI) staining. **c**, Quantification of interphasic and mitotic K562 cells after nocodazole treatment and double fixation. **d**, Gating strategy to sort MPM-2 positive nocodazole treated K562 cells after double fixation. Mitotic cell purity after sorting is shown. **e**, Gene tracks for GATA2 binding sites at *CD9 locus*. Bookmarked sites are highlighted in grey. **f**, Gene Ontology (GO) biological processes (BP) for the top 1,000 gene-related peaks in non-bookmarked genes in asynchronous cells (Async only), mitotic (Mit) and mitotic clusters 1 (C1) and 2 (C2). Categories that contain more than 5 peaks per category are displayed. Coloured scale represents the adjusted *p*-value and the circle size the number of peaks per group. **g,** Motif discovery analysis for GATA2 target sites per group of peaks. Coloured scale represents the adjusted *p*-value and the circle size the percentage of peaks containing a particular motif. **h**, Gene body distribution of Mit and Async only GATA2 binding sites.

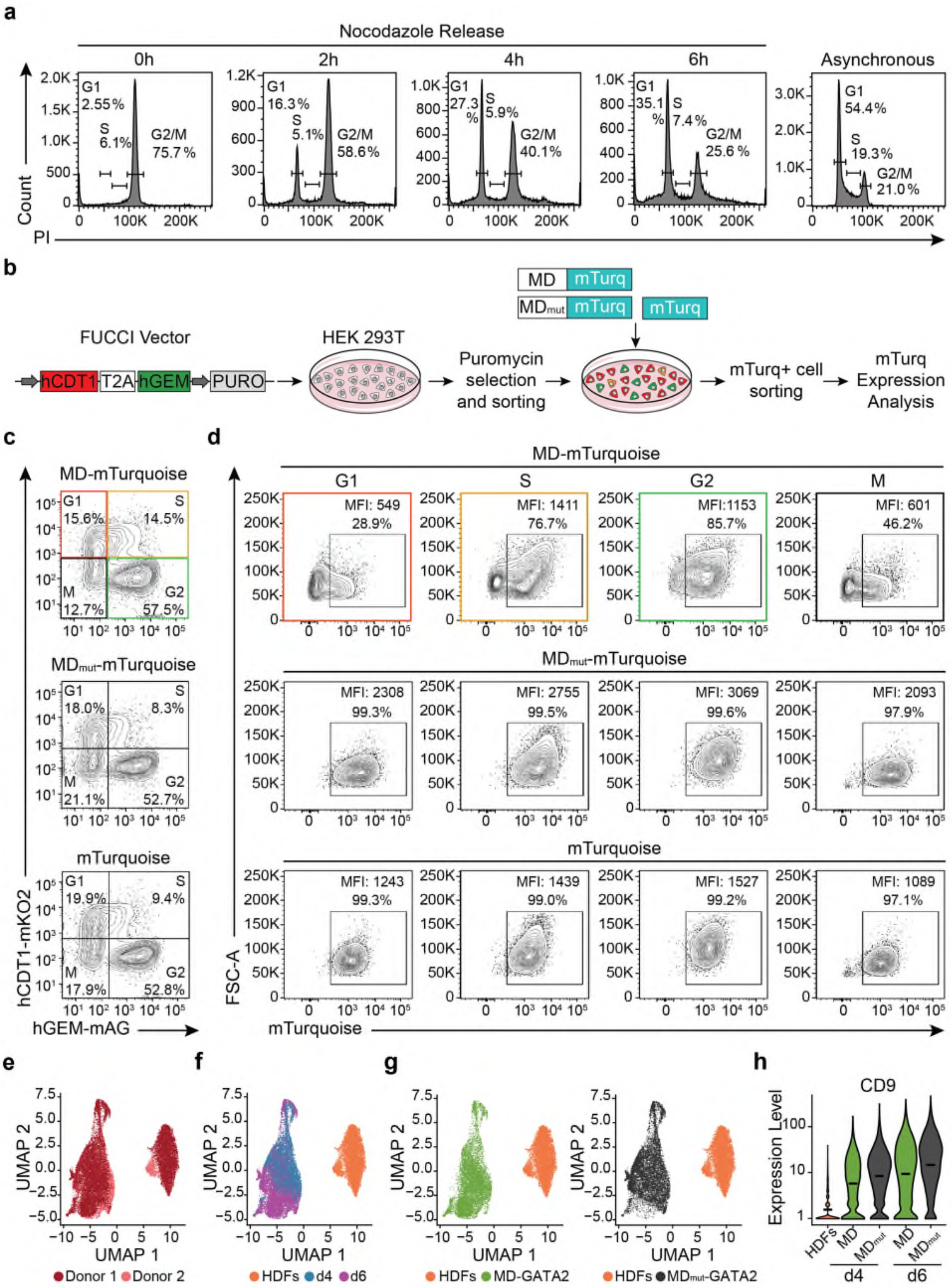

Supplementary Fig. 5. **Protein degradation driven by the mitotic degradation (MD) domain of cyclin B1 delays hemogenic reprogramming. a,** Cell cycle analysis of asynchronous and

nocodazole treated HEK 293T cells before (0h) and 2, 4 and 6 hours (h) after release from nocodazole arrest. PI – propidium iodide**.** Percentage of cells in G1, S and G2/M phases of the cell cycle are indicated. **b**, Experimental strategy to assess mTurquoise (mTurq) protein degradation when fused to the MD domain or a mutated non-functional version (MD_{mut}). HEK 293T cell line stably expressing a fluorescent ubiquitination-based cell cycle indicator (FUCCI) vector was generated by transduction with a FUCCI lentiviral vector, encoding a puromycin (Puro) resistance gene. After puromycin selection and purification of GFP+ cells by FACS, cells were transduced with lentiviral vectors encoding MD-mTurquoise, MD_{mut}-mTurquoise or mTurquoise. mTurquoise+ cells were FACS sorted, plated and mTurquoise expression was measured 48 hours later by flow cytometry. hCDT1 – human CDT1 fused to mKusabira-Orange2 (mKO2). hGEM – human Geminin fused to mAzamiGreen (mAG). **c**, Flow cytometry plots showing the separation of cell cycle populations: interphase – G1 (red – mKO2+mAG-), S (yellow – mKO2+mAG+) and G2 (green – mKO2-mAG+), and mitosis – M (black – mKO2-mAG-) of cells expressing MD/MD_{mut}-mTurquoise or mTurquoise alone. **d**, Flow cytometry plots showing mTurquoise expression gated within each cell cycle phase population. MFI – mean fluorescence intensity. **e-g**, Uniform Manifold Approximation and Projection (UMAP) analysis of 32,773 single transcriptomes of FACS sorted, live (Dapi-) human dermal fibroblasts (HDFs) and HDFs undergoing hemogenic reprogramming with overexpression of GFI1B, FOS and MD-GATA2 or GFI1B, FOS and MD_{mut}-GATA2 from two donors. (**e**) shows single cells coloured by donor (Donor 1 and Donor 2) (**f**) highlights untransduced HDFs and reprogrammed cells at day (d) 4 and d6 and (**g**) shows transduced cells with either MD-GATA2 (left) or MD_{mut}-GATA2 (right). **h**, Expression levels of the *CD9* gene in untransduced HDFs and in MD-GATA2 or MD_{mut}-GATA2 transduced cells at d4 and d6 of hemogenic reprogramming. Mean is represented.

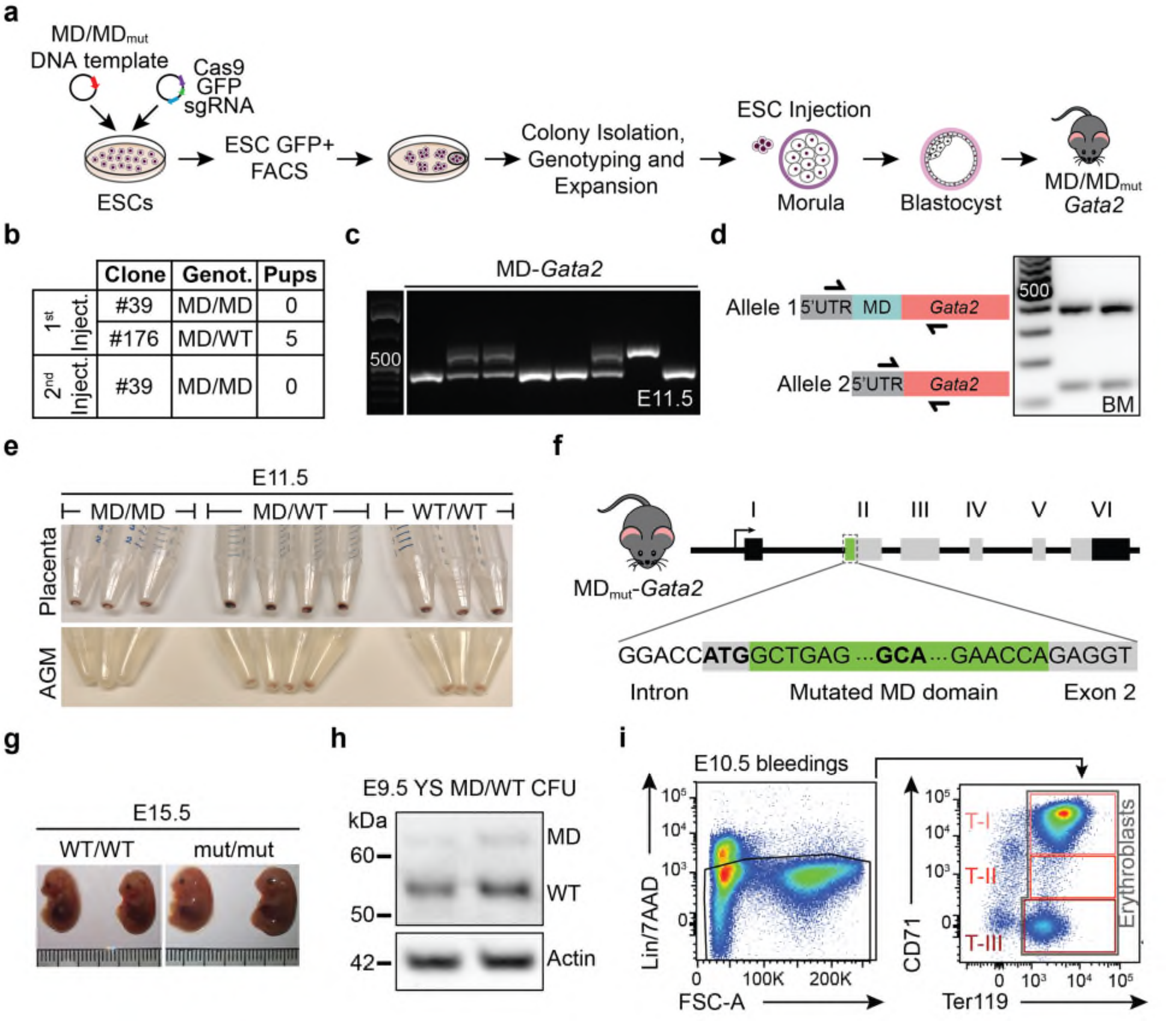

Supplementary Fig. 6. Characterisation of MD- and MD$_{mut}$-*Gata2* mouse models. a, Strategy to generate a mouse model to assess the role of mitotic bookmarking by GATA2 *in vivo*. The mitotic degradation (MD) domain of cyclin B1 and a mutated non-functional domain (MD$_{mut}$) template sequences were delivered in a pMX plasmid to mouse embryonic stem cells (ESCs) together with a pX458-GFP vector containing a single guide (sg) RNA for *Gata2* start codon region. GFP+ cells were FACS sorted, and individual clones were isolated, genotyped, expanded and injected into mouse morulae to generate both MD-*Gata2* and MD$_{mut}$-*Gata2* models. **b**, Number of pups obtained after two independent morula injections with homozygous or heterozygous ESC clones for the MD insert. **c**, Genotyping of embryonic day (E) 11.5 embryos after crossing heterozygous MD-*Gata2* mice. A 630 base pairs band indicates the presence of the

insert while a lower band of 400 bps represents the wild-type (WT) *Gata2* allele. Genotyping was performed in individual embryos or adult mice before each experiment. **d**, Gene expression of the MD and *Gata2* WT alleles. mRNA of sorted LSK cells from bone marrow (BM) heterozygous mice were isolated, reverse transcribed and amplified by PCR. The stronger, heavier band corresponds to the MD sequence. Experiment was performed once with cells from 3 different adult heterozygous mice. Representative results for two mice are shown. **e**, Representative pictures of placenta pellets and caudal regions isolated from single E11.5 embryos after tissue dissociation. **f**, Scheme showing the insertion of the MD_{mut} domain after the ATG of the *Gata2* gene in exon 2. **g**, Representative images of MD_{mut}-*Gata2* embryos at E15.5. Scale bar = 1 mm. **h**, Western blot analysis of heterozygous MD-GATA2 E9.5 yolk sac-derived hematopoietic colonies after 6 days of culture. Bands corresponding to MD-GATA2 (MD) and wild-type (WT) proteins are shown. Experiment was performed once with cells from 4 different embryos. Representative results for two embryos are shown. kDA, kilodaltons. **i**, Representative flow cytometry plots of erythroblast quantification after whole embryo bleeding to assess erythroid output. MD-*Gata2* embryonic blood was stained with lineage antibodies (B220/CD3ε/Mac1/Gr1) and the erythroblast markers CD71 and Ter119. Live lineage negative cells (Lin-7AAD-) were gated into three types of erythroblasts from immature to more mature red blood cells (Type I-III) according to the expression of CD71 and Ter119. The grey gate includes all erythroblasts.

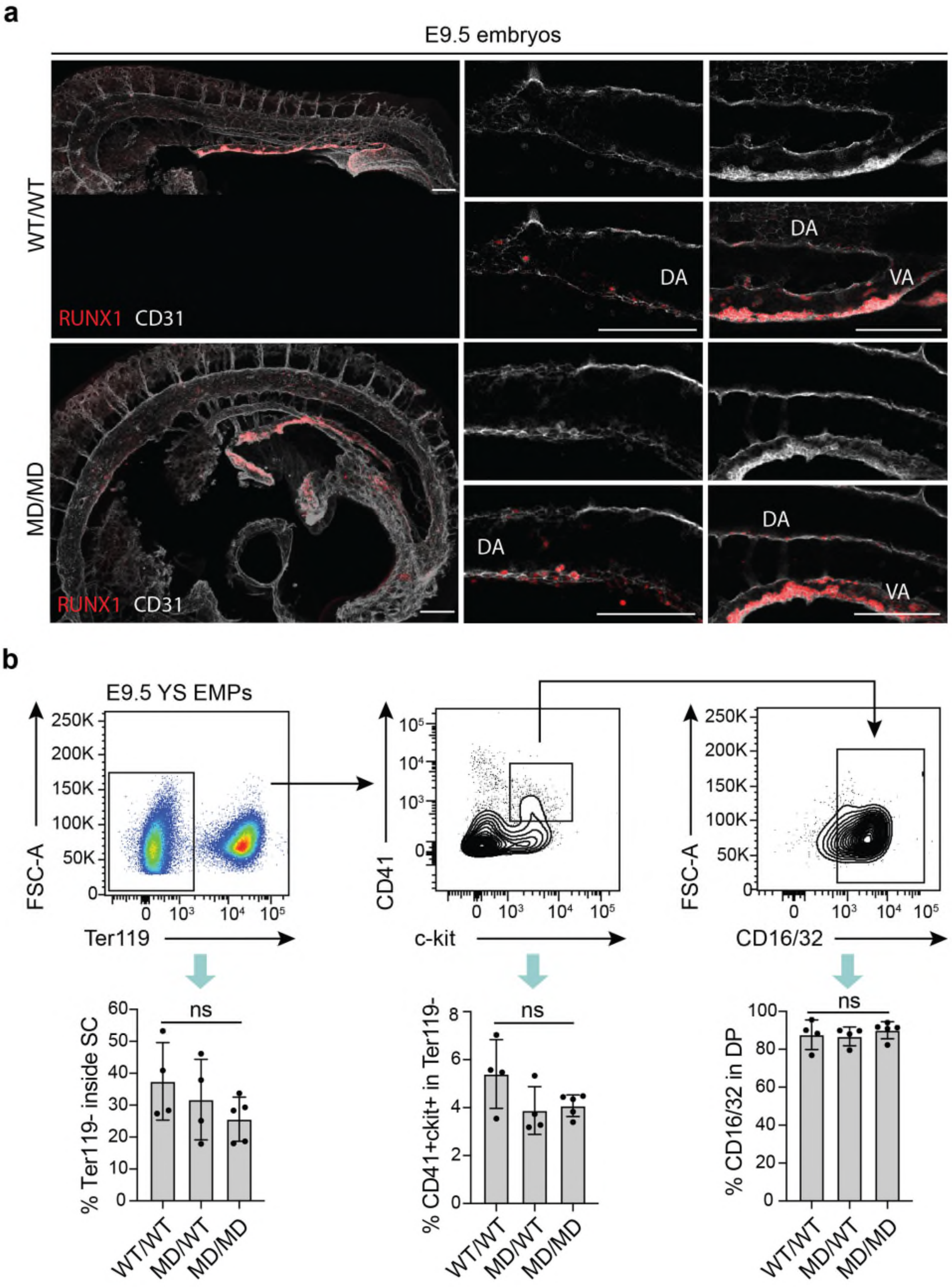

Supplementary Fig. 7. Haematopoiesis is not impaired in MD-*Gata2* homozygous embryos at embryonic day 9.5. a, Immunohistochemistry images of whole mounted E9.5 WT and MD

homozygous embryos showing vasculature expressing RUNX1 (red) and CD31 (white), highlighting the dorsal aorta (DA) and vitelline arteries (VA). Scale bars = 100 µm. **b**, E9.5 yolk sac (YS) erythro-myeloid progenitor (EMPs) staining. Yolk sacs from E9.5 embryos were isolated, dissociated and stained with Ter119, c-kit, FITC-CD41 and CD16/32. EMPs were defined as Ter119-CD41+c-kit+CD16/32+. Representative plots with gating strategy (left) and percentage of each respective cell population (right) are shown (n=4). SC – single cells. DP – double positive. Mean±SD is represented. Statistical significance was analysed by one-way ANOVA followed by Bonferroni's multiple comparison test. ns – nonsignificant.

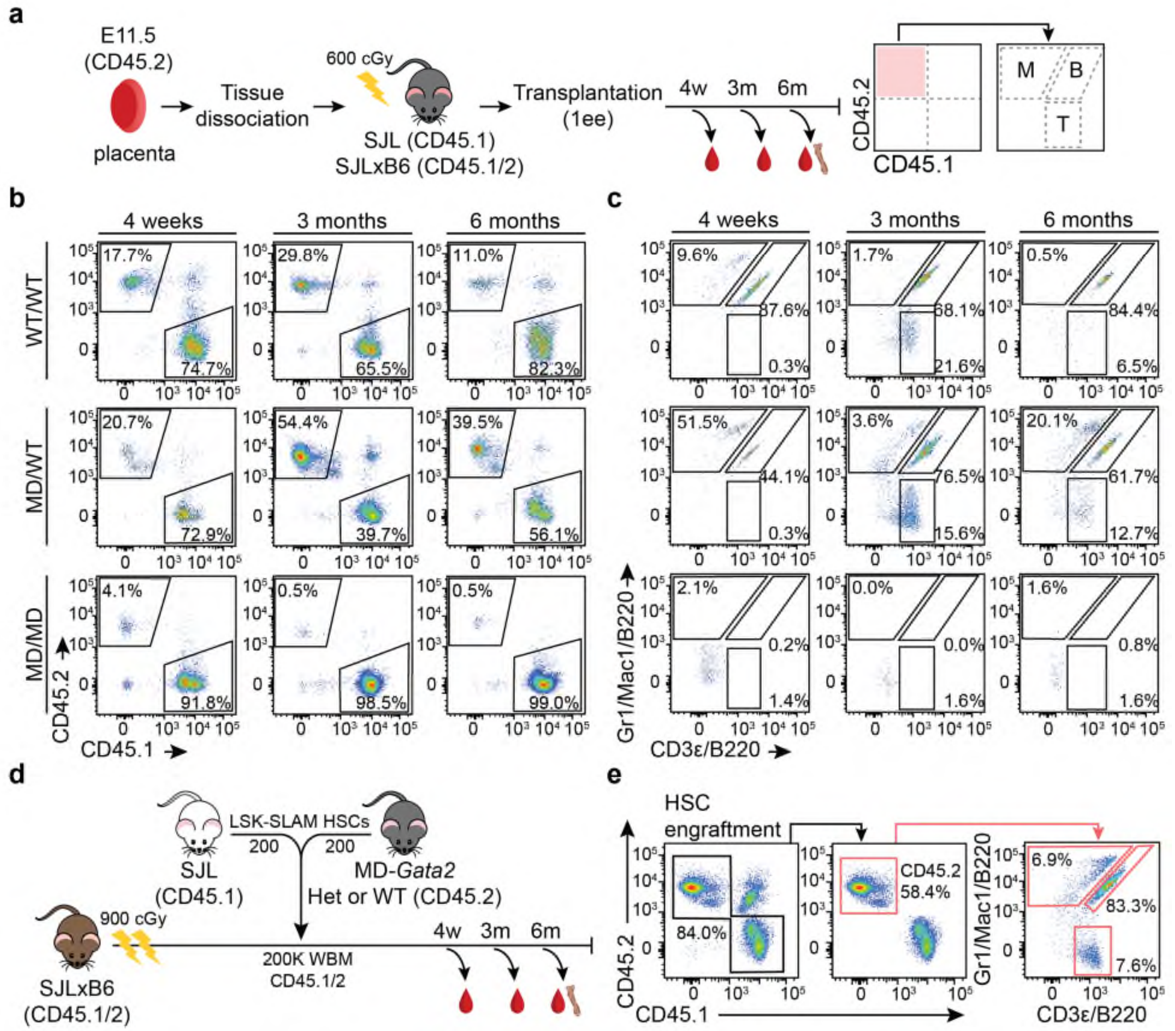

Supplementary Fig. 8. Embryonic and adult transplantation strategies to assess the role of GATA2 at mitosis-to-G1 transition for HSPC generation and maintenance. a, E11.5 placentas were isolated, dissociated and transplanted as one embryo equivalent (ee) into sub-lethally irradiated B6.SJL (CD45.1) or C57BL/6JxB6.SJL (CD45.1/2) mice. Mice were bled 4 weeks (w), 3 months (m), and 6 months after transplantation to assess donor engraftment (CD45.2+) and contribution to myeloid (M), B-cell (B) and T-cell (T) lineages. Hosts' bone marrow cells from the left leg and ilium bones were isolated at the experimental end-point of 6 months and analysed in a similar manner. **b**, Representative flow cytometry plots with the gating strategy to evaluate donor chimerism (CD45.2+). Only mice with donor chimerism above the 1% threshold were considered in this analysis. **c**, Gating strategy for assessing the percentage of myeloid

(Gr1+Mac1+), B- (B220+) and T- (CD3ε+) donor-derived cells. **d**, Competitive transplantation strategy to address function of adult bone marrow HSCs. Two hundred Lineage^{-}Sca-1^{+}Kit^{+}CD150^{+}CD48^{-} LSK-SLAM HSCs from a competitor CD45.1 SLJ mouse or from MD/WT or WT/WT MD-*Gata2* mice were FACS purified, mixed 1:1 and injected into lethally irradiated CD45.1/2 hosts, together with 200,000 support whole bone marrow (WBM) cells. Blood was collected for analysis 4w, 3m and 6m after transplantation to assess donor engraftment (CD45.2+) and contribution to myeloid, B-cell and T-cell lineages. Recipients' bone marrow was analysed at the experimental end-point of 6 months. **e**, Representative gating strategy to evaluate donor chimerism (CD45.2+) and for assessing the percentage of myeloid (Gr1+Mac1+), B- (B220+) and T- (CD3ε+) donor-derived cells.

Western Blots and Gels

Supplementary Figure 1b

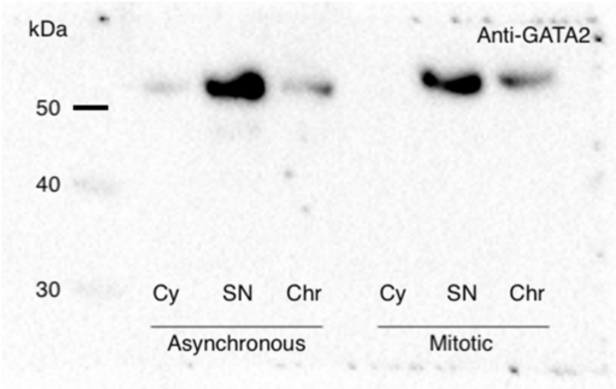

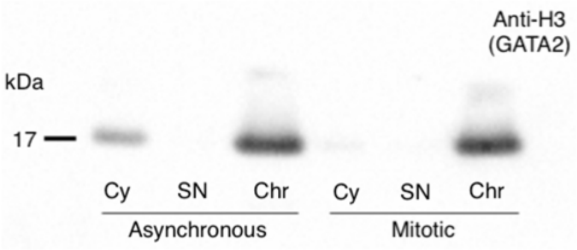

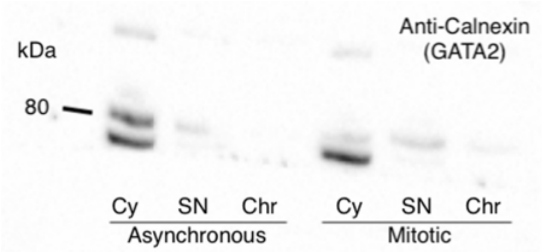

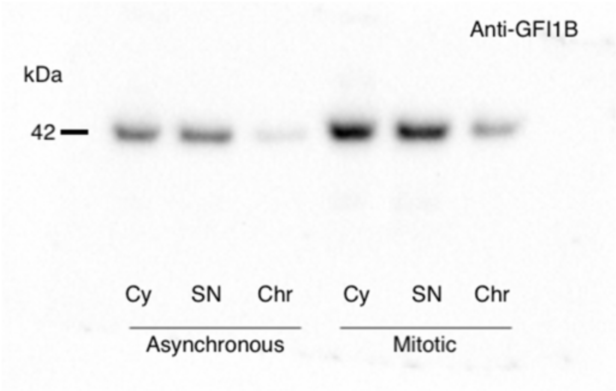

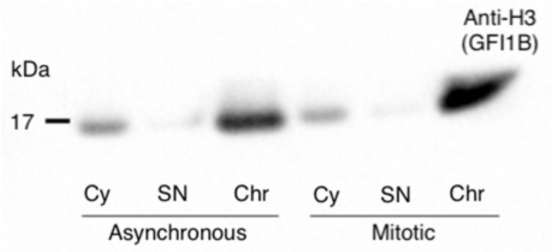
Anti-H3
(GFI1B)
kDa
17
Cy
SN
Chr
Cy
SN
Chr
Asynchronous
Mitotic

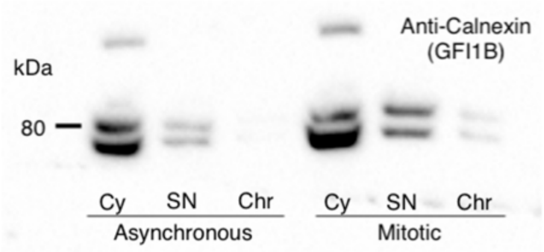
Anti-Calnexin
(GFI1B)
kDa
80
Cy
SN
Chr
Cy
SN
Chr
Asynchronous
Mitotic

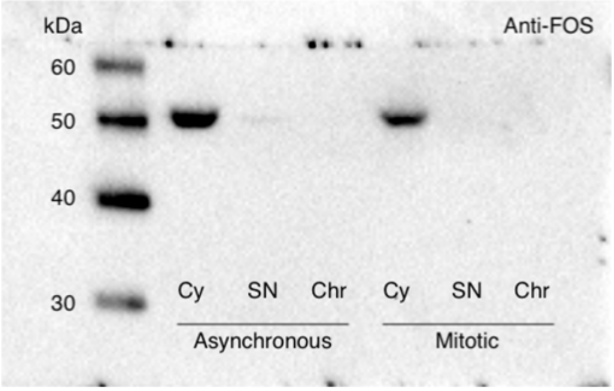
kDa
Anti-FOS
60
50
40
30
Cy
SN
Chr
Cy
SN
Chr
Asynchronous
Mitotic

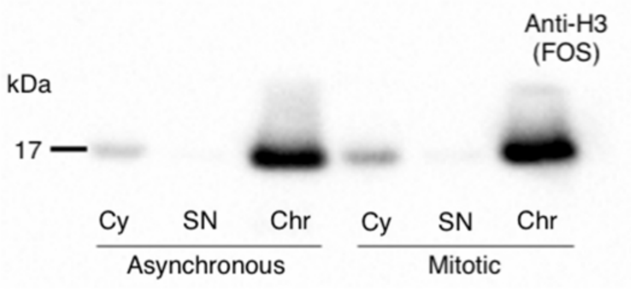
Anti-H3
(FOS)
kDa
17
Cy
SN
Chr
Cy
SN
Chr
Asynchronous
Mitotic

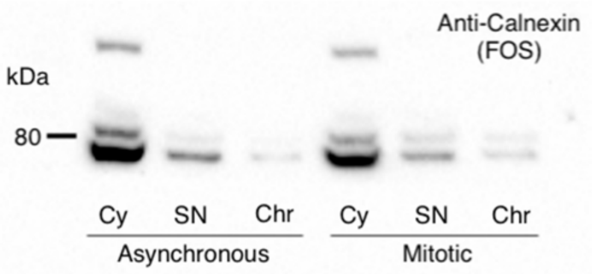
Anti-Calnexin
(FOS)
kDa
80
Cy
SN
Chr
Cy
SN
Chr
Asynchronous
Mitotic

Supplementary Figure 2a

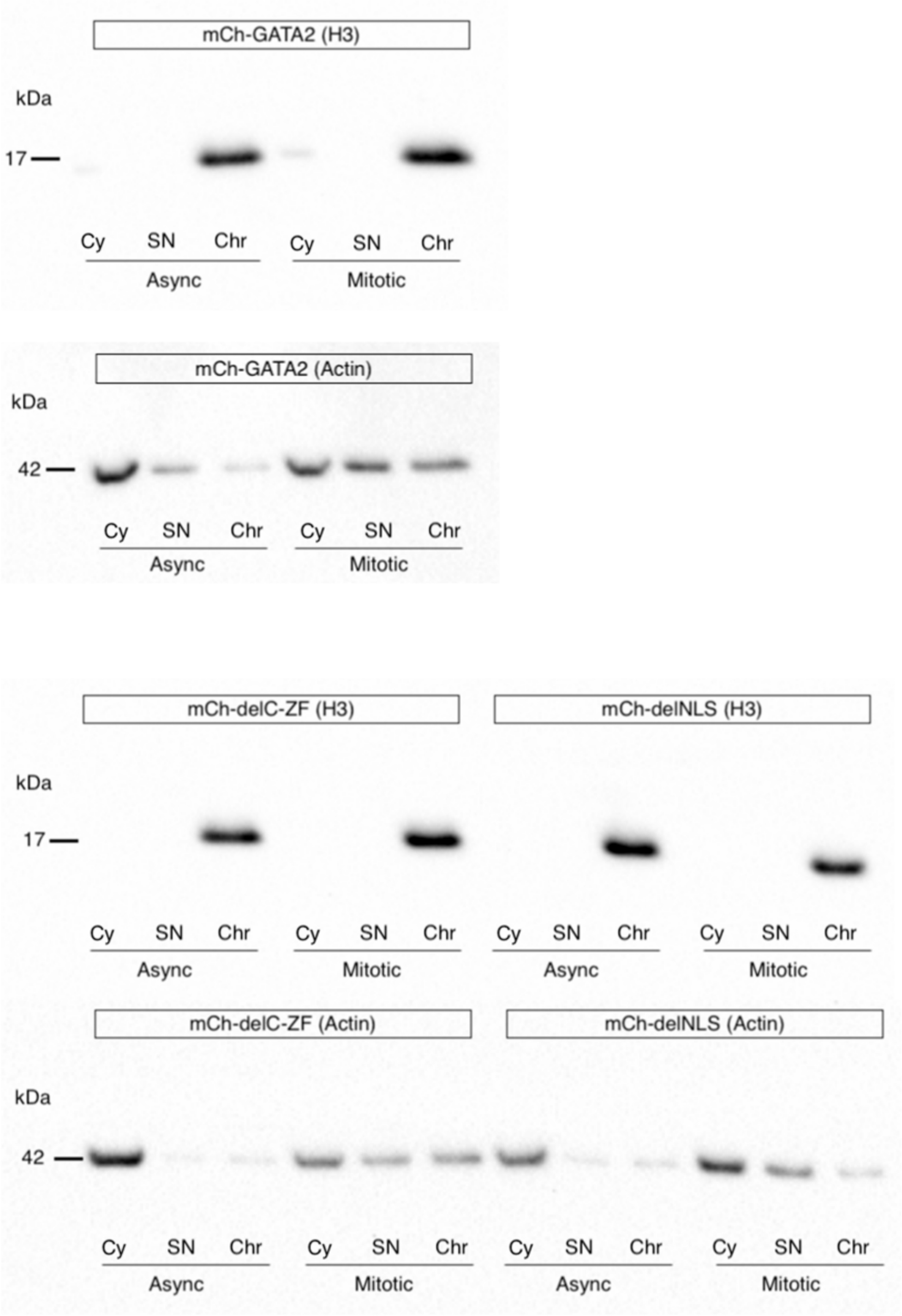

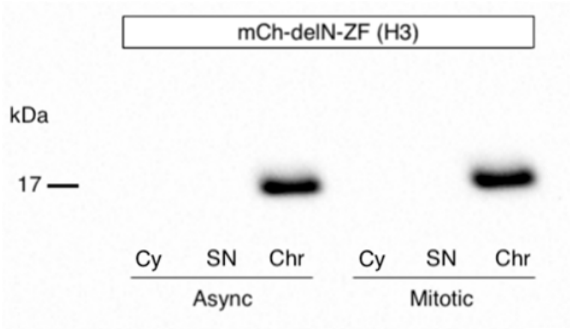
mCh-delN-ZF (H3)
kDa
17
Cy
SN
Chr
Cy
SN
Chr
Async
Mitotic

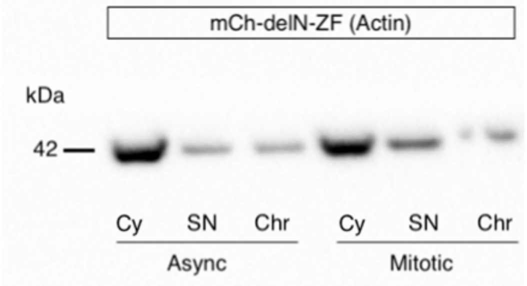
mCh-delN-ZF (Actin)
kDa
42
Cy
SN
Chr
Cy
SN
Chr
Async
Mitotic

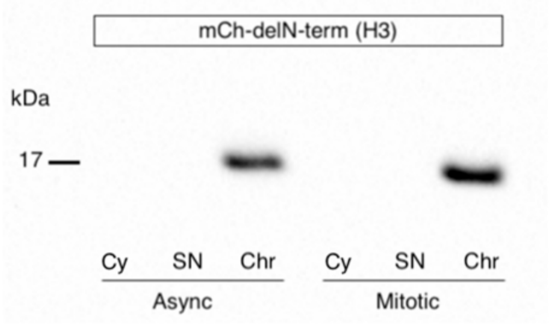
mCh-delN-term (H3)
kDa
17
Cy
SN
Chr
Cy
SN
Chr
Async
Mitotic

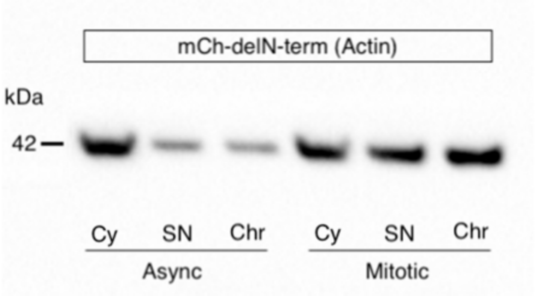
mCh-delN-term (Actin)
kDa
42
Cy
SN
Chr
Cy
SN
Chr
Async
Mitotic

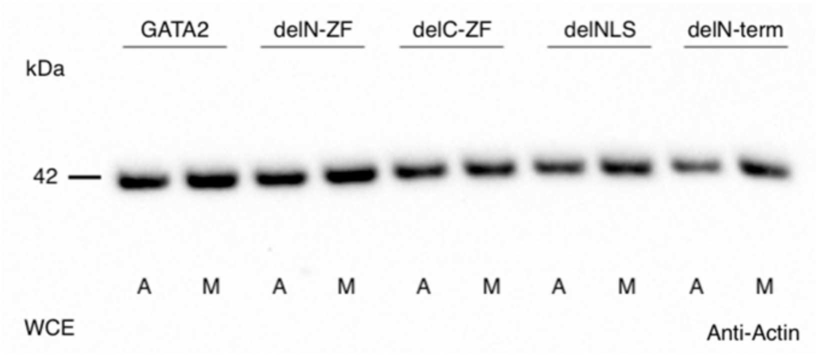
GATA2
delN-ZF
delC-ZF
delNLS
delN-term
kDa
42
A
M
A
M
A
M
A
M
A
M
WCE
Anti-Actin

Supplementary Figure 2b

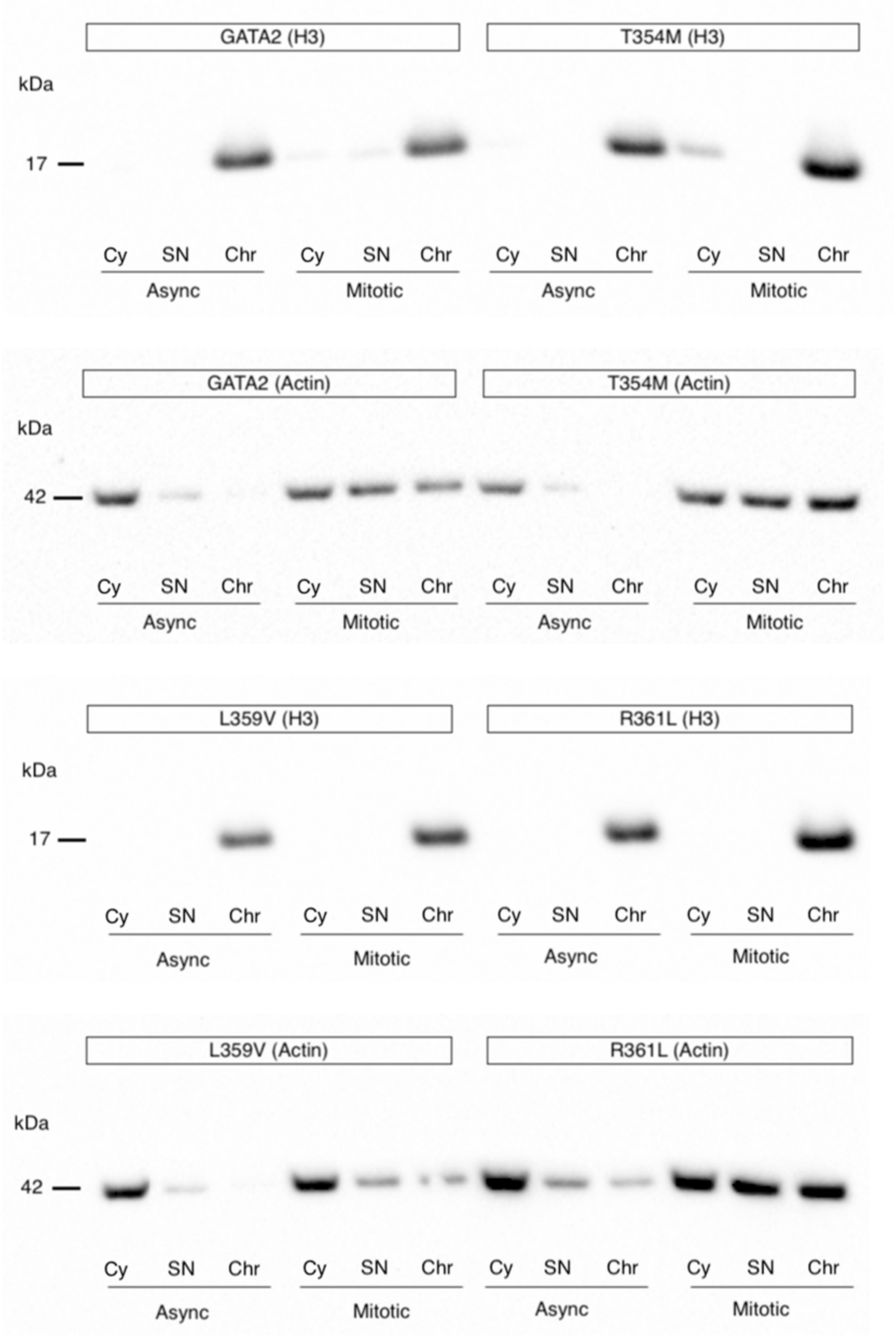

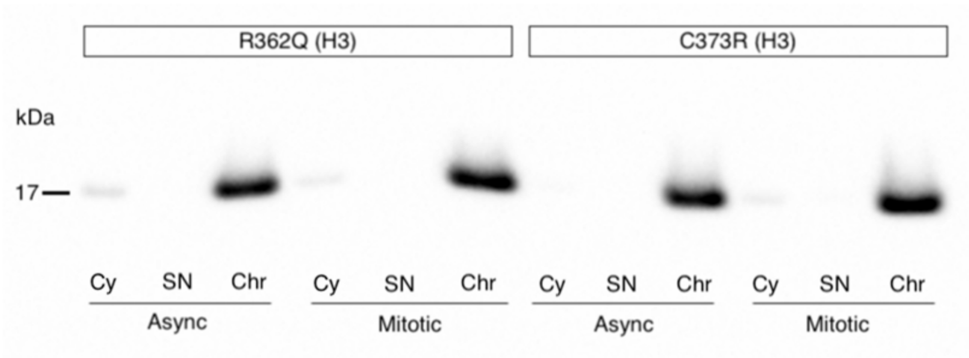
R362Q (H3)
C373R (H3)
kDa
17
Cy SN Chr Cy SN Chr Cy SN Chr Cy SN Chr
Async
Mitotic
Async
Mitotic

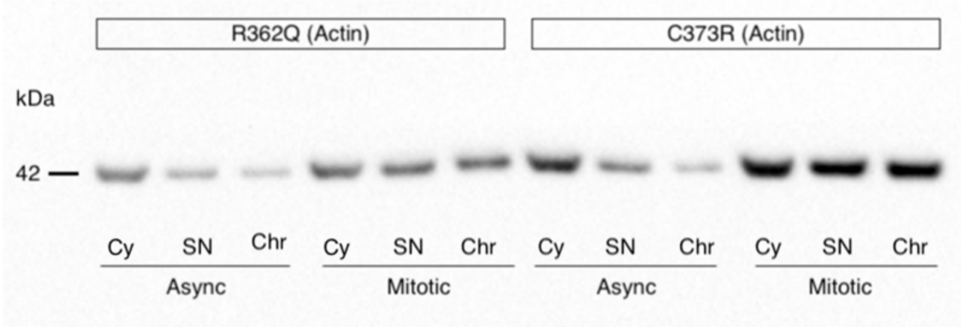
R362Q (Actin)
C373R (Actin)
kDa
42
Cy SN Chr Cy SN Chr Cy SN Chr Cy SN Chr
Async
Mitotic
Async
Mitotic

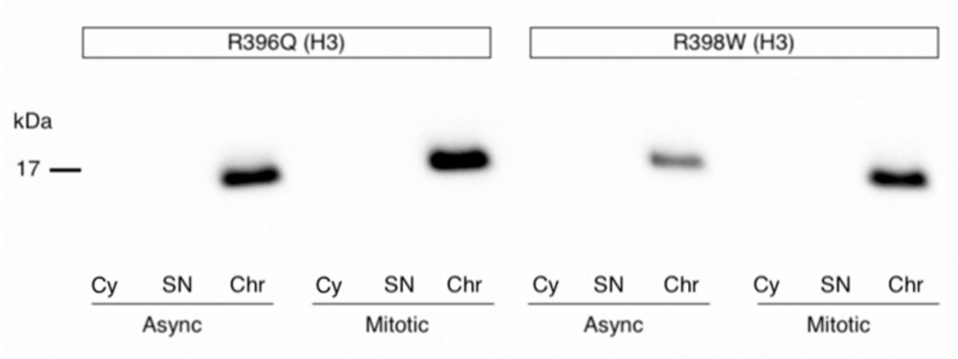
R396Q (H3)
R398W (H3)
kDa
17
Cy SN Chr Cy SN Chr Cy SN Chr Cy SN Chr
Async
Mitotic
Async
Mitotic

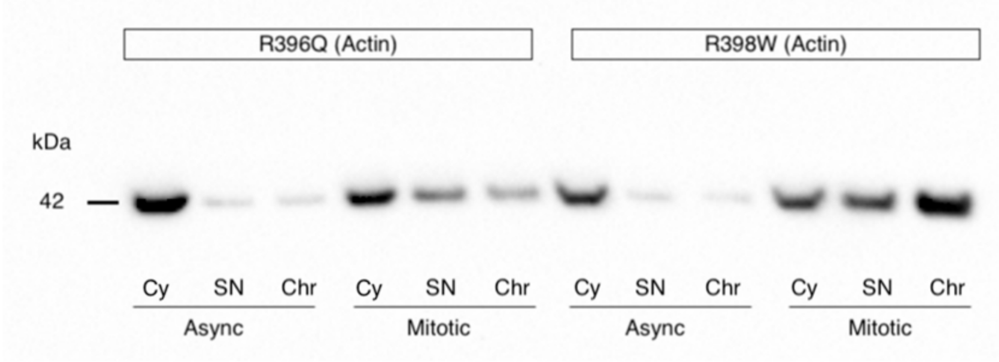
R396Q (Actin)
R398W (Actin)
kDa
42
Cy SN Chr Cy SN Chr Cy SN Chr Cy SN Chr
Async
Mitotic
Async
Mitotic

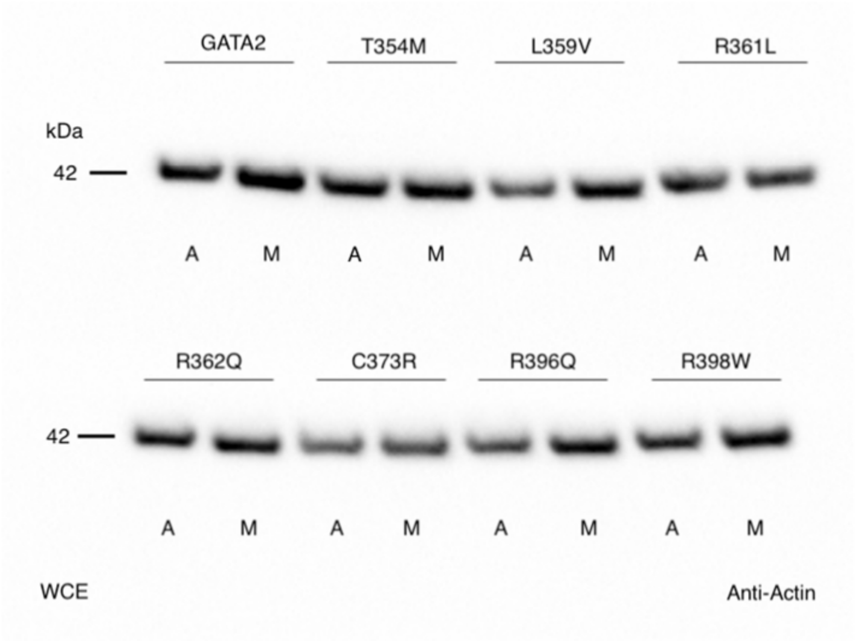

Supplementary Figure 6c

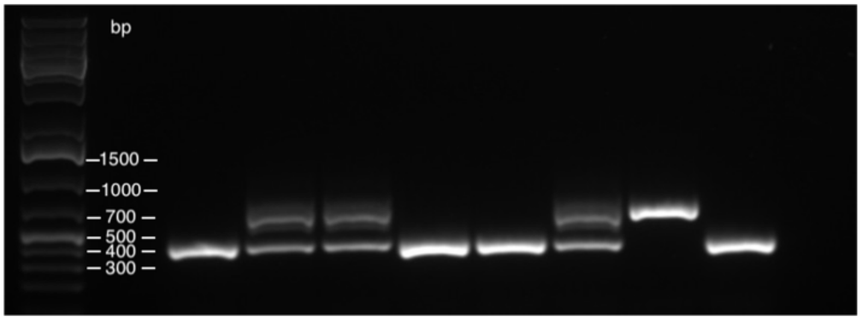

Supplementary Figure 6d

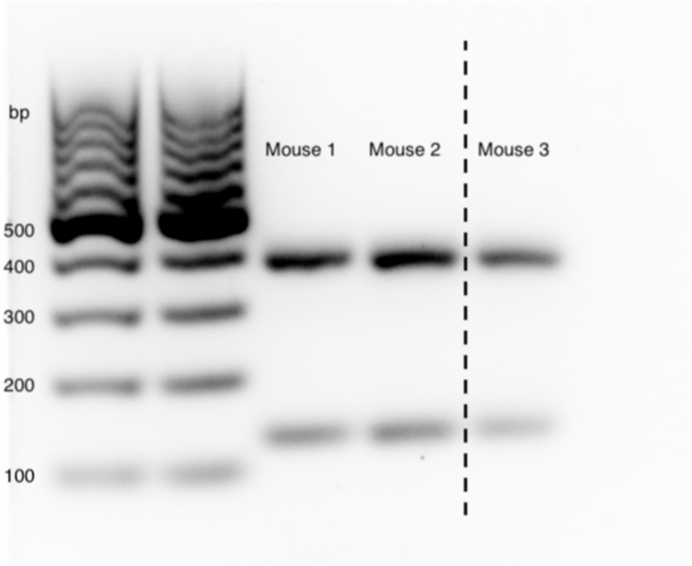

Supplementary Figure 6h

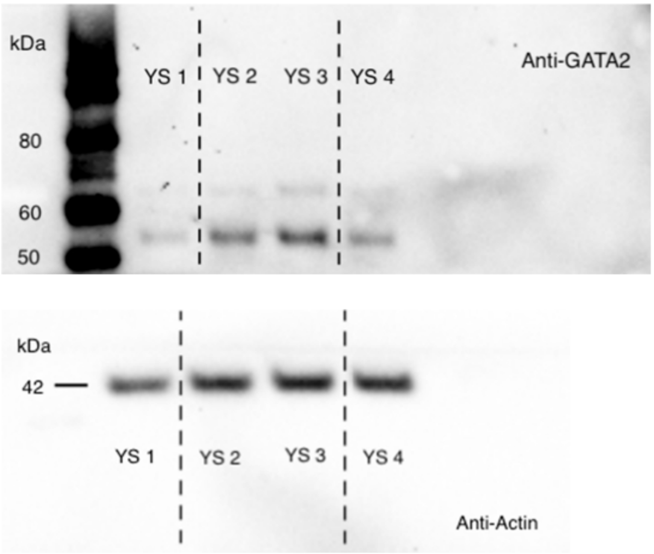

Supplement: Supplementary file 1 — Supplementary Information [file 41467_2023_40391_MOESM1_ESM.pdf]
