# Supplementary material for: GATA2 mitotic bookmarking is required for definitive haematopoiesis
Source: Nat Commun. 2023 Aug 14;14:4645. doi: 10.1038/s41467-023-40391-x (PMC10425459; doi:10.1038/s41467-023-40391-x)
Supplement: Supplementary file 3 — Description of Additional Supplementary Files [file 41467_2023_40391_MOESM3_ESM.pdf]

Description of Additional Supplementary Files

Supplementary Data 1

Plasmids, primers, antibodies and fluorescent dyes used in this study, as well as DNA template sequences for MD and MD_{mut} domain insertion upstream *Gata2* gene via CRISPR/Cas9.

Supplementary Data 2

ChIP-seq data analysis including gene lists for asynchronous (A), mitotic (M) and bookmarked genes (A-M intersection); ChromHMM enrichment values, *de novo* motif discovery for GATA2 in asynchronous and mitotic K562 cells; GREAT Enrichment GO BP terms for the peaks associated with the top 1000 genes; GEO accession codes from publicly available ChIP-seq, ATAC-seq and DNase-seq data.

Supplementary Data 3

ScRNA-seq data analysis including information regarding cell samples; Differential gene expression analysis at day 4 and 6 of reprogramming with MD-GATA2 or MD_{mut}-GATA2 and integration with ChIP-seq data; ChIP Enrichment Analysis (ChEA) table with differentially expressed genes. For differential expression analysis between cell states, we used Seurat v4 FindMarkers function using two-sided Wilcoxon Rank Sum test with logfc.threshold = 0.1, min.pct = 0.25 and adjusted p-value, based on bonferroni correction <0.05.

Supplementary Movie 1. GATA2 is retained in condensed chromatin during all phases of mitosis in human dermal fibroblasts (HDFs). HDFs were co-transduced with mCherry-GATA2 fusion protein and H2B-mTurquoise and followed by time-lapse fluorescence live-cell imaging for over 24h. Cell morphology (phase-contrast) changes and DNA (blue signal from H2B-mTurquoise) condenses as representative cell undergoes mitosis. Simultaneously, GATA2 (red signal from mCherry) remains bound to condensed chromatin as cell divide. Pictures were taken every 10 minutes and time-lapse was clipped to show 1 cell division in a 4 frames per second video. Scale bar, 10 μm.

Supplementary Movie 2. GFI1B gets enriched at mitotic chromatin during anaphase in human dermal fibroblasts (HDFs). HDFs were co-transduced with mCherry-GFI1B fusions and H2B-mTurquoise and followed by time-lapse fluorescence live-cell imaging for over 24h. Phasecontrast denotates cell morphology changes and blue signal from H2B-mTurquoise shows DNA condensation during mitosis. As the representative cell divides, mCherry-GFI1B (red signal) disperses through the cytoplasm and gets enriched in condensed at anaphase-telophase. Pictures were taken every 10 minutes and time-lapse was clipped to show 1 cell division in a 4 frames per second video. Scale bar, 10 μm.

Supplementary Movie 3. FOS is excluded from chromatin during mitosis in human dermal fibroblasts (HDFs). HDFs were co-transduced with mCherry-FOS fusion proteins and H2BmTurquoise and followed by time-lapse fluorescence live-cell imaging for over 24h. As DNA in blue (H2B-mTurquoise) condenses and cell morphology (phase-contrast) changes, mCherryFOS is evicted from chromatin until the next interphase, when nuclear envelop is re-established. Pictures were taken every 10 minutes and time-lapse was clipped to show 1 cell division in a 4 frames per second video. Scale bar, 10 µm.

Supplementary Movie 4. GATA2 mutation C373R impairs GATA2 retention during mitosis. HEK 293T were co-transduced with mCherry-C373R fusion protein and H2B-mTurquoise and followed by time-lapse fluorescence live-cell imaging for over 24h. The GATA2 mutant C373R (red signal from mCherry) disperses through the cytoplasm as DNA (blue signal from H2BmTurquoise) condenses and representative cell (phase-contrast) undergoes cell division. Pictures were taken every 5 minutes and time-lapse was clipped to show 1 cell division in a 4 frames per second video. Scale bar, 10 µm.

Supplementary Movie 5. GATA2 mutation L359V does not impair GATA2 retention during mitosis. HEK 293T were co-transduced with mCherry-L359V fusion protein and H2BmTurquoise and followed by time-lapse fluorescence live-cell imaging for over 24h. The GATA2 mutant L359V (red signal from mCherry) remains bound to chromatin (blue signal from H2BmTurquoise) as representative cell (phase-contrast) undergoes mitosis. Pictures were taken every 5 minutes and time-lapse was clipped to show 1 cell division in a 4 frames per second video. Scale bar, 10 µm.
