# Supplementary material for: GATA2 mitotic bookmarking is required for definitive haematopoiesis
Source: Nat Commun. 2023 Aug 14;14:4645. doi: 10.1038/s41467-023-40391-x (PMC10425459; doi:10.1038/s41467-023-40391-x)
Supplement: Supplementary file 12 — Reporting Summary [file 41467_2023_40391_MOESM12_ESM.pdf]

nature portfolio

Corresponding author(s): Carlos-Filipe Pereira

Last updated by author(s): Jul 7, 2023

Reporting Summary

Nature Portfolio wishes to improve the reproducibility of the work that we publish. This form provides structure for consistency and transparency in reporting. For further information on Nature Portfolio policies, see our Editorial Policies and the Editorial Policy Checklist.

Statistics

For all statistical analyses, confirm that the following items are present in the figure legend, table legend, main text, or Methods section.

n/a	Confirmed	
☐	☒	The exact sample size (*n*) for each experimental group/condition, given as a discrete number and unit of measurement
☐	☒	A statement on whether measurements were taken from distinct samples or whether the same sample was measured repeatedly
☐	☒	The statistical test(s) used AND whether they are one- or two-sided<br>*Only common tests should be described solely by name; describe more complex techniques in the Methods section.*
☒	☐	A description of all covariates tested
☐	☒	A description of any assumptions or corrections, such as tests of normality and adjustment for multiple comparisons
☐	☒	A full description of the statistical parameters including central tendency (e.g. means) or other basic estimates (e.g. regression coefficient) AND variation (e.g. standard deviation) or associated estimates of uncertainty (e.g. confidence intervals)
☐	☒	For null hypothesis testing, the test statistic (e.g. *F*, *t*, *r*) with confidence intervals, effect sizes, degrees of freedom and *P* value noted<br>*Give P values as exact values whenever suitable.*
☒	☐	For Bayesian analysis, information on the choice of priors and Markov chain Monte Carlo settings
☒	☐	For hierarchical and complex designs, identification of the appropriate level for tests and full reporting of outcomes
☒	☐	Estimates of effect sizes (e.g. Cohen's *d*, Pearson's *r*), indicating how they were calculated

Our web collection on statistics for biologists contains articles on many of the points above.

Software and code

Policy information about availability of computer code

Data collection	Zeiss ZEN 3.1 (blue); BD FACSDiva (v9.0); Image Lab (v5.2.1) ; Illumina NextSeq 500 system; ZEN 2011 LSM (black)
Data analysis	FLOWJO LLC (v10.6.1); GraphPad Prism (v9); ImageJ (v2.1.0) ; BWA (v0.7.12); samtools (v1.12); MACS (v2.1.0); Genrich (v0.6.1); bedtools (v2.30); deeptools (v2.5.4); base R (v4.0.3); ChIPpeakAnno R library (v3.24.2); ChIPseeker R library (v1.26.2); Cyclebase 3.0; Cell Ranger (v6.0.1); Seurat R library (v4.1.0); FIMO (MEME Suite v5.5.0); GREAT (v4.0.4); LinReg PCR software (v2021.2)

For manuscripts utilizing custom algorithms or software that are central to the research but not yet described in published literature, software must be made available to editors and reviewers. We strongly encourage code deposition in a community repository (e.g. GitHub). See the Nature Portfolio guidelines for submitting code & software for further information.

Data

Policy information about availability of data

All manuscripts must include a data availability statement. This statement should provide the following information, where applicable:

- Accession codes, unique identifiers, or web links for publicly available datasets
- A description of any restrictions on data availability
- For clinical datasets or third party data, please ensure that the statement adheres to our policy

Data supporting this work is available upon request. Source data are provided with this paper. ChIP-seq and scRNA-seq data has been deposited in the Gene Expression Omnibus (GEO) with accession codes GSE207551 (https://www.ncbi.nlm.nih.gov/geo/query/acc.cgi?acc=GSE207551), and GSE221691 (https://www.ncbi.nlm.nih.gov/geo/query/acc.cgi?acc=GSE221691), respectively. Processed data was mapped to the human genome assembly (hg38, GRCh38.p14). Public

datasets used in this study can be accessed under accession numbers GSM733680 (https://www.ncbi.nlm.nih.gov/geo/query/acc.cgi?acc=GSM733680), GSM733656 (https://www.ncbi.nlm.nih.gov/geo/query/acc.cgi?acc=GSM7336569), GSM733658 (https://www.ncbi.nlm.nih.gov/geo/query/acc.cgi?acc=GSM733658), GSM733692 (https://www.ncbi.nlm.nih.gov/geo/query/acc.cgi?acc=GSM733692), GSM733714 (https://www.ncbi.nlm.nih.gov/geo/query/acc.cgi?acc=GSM733714), GSM733776 (https://www.ncbi.nlm.nih.gov/geo/query/acc.cgi?acc=GSM733776), GSM733778 (https://www.ncbi.nlm.nih.gov/geo/query/acc.cgi?acc=GSM733778), GSM733651 (https://www.ncbi.nlm.nih.gov/geo/query/acc.cgi?acc=GSM733651), GSM733675 (https://www.ncbi.nlm.nih.gov/geo/query/acc.cgi?acc=GSM733675), GSM733786 (https://www.ncbi.nlm.nih.gov/geo/query/acc.cgi?acc=GSM733786), GSM733653 (https://www.ncbi.nlm.nih.gov/geo/query/acc.cgi?acc=GSM733653), GSM733777 (https://www.ncbi.nlm.nih.gov/geo/query/acc.cgi?acc=GSM733777), GSM803540 (https://www.ncbi.nlm.nih.gov/geo/query/acc.cgi?acc=GSM803540), GSE96253 (https://www.ncbi.nlm.nih.gov/geo/query/acc.cgi?acc=GSE96253), GSM803384 (https://www.ncbi.nlm.nih.gov/geo/query/acc.cgi?acc=GSM803384), GSM777644 (https://www.ncbi.nlm.nih.gov/geo/query/acc.cgi?acc=GSM777644), GSM1010820 (https://www.ncbi.nlm.nih.gov/geo/query/acc.cgi?acc=GSM1010820), GSE170378 (https://www.ncbi.nlm.nih.gov/geo/query/acc.cgi?acc=GSE170378), GSE172523 (https://www.ncbi.nlm.nih.gov/geo/query/acc.cgi?acc=GSE172523) for Histones ChIP-seq, TFs ChIP-seq, DNAse-seq and ATAC-seq in K562 from ENCODE project; GSM1278240 (https://www.ncbi.nlm.nih.gov/geo/query/acc.cgi?acc=GSM1278240), GSM1278241 (https://www.ncbi.nlm.nih.gov/geo/query/acc.cgi?acc=GSM1278241), GSM1278242 (https://www.ncbi.nlm.nih.gov/geo/query/acc.cgi?acc=GSM1278242) for TFs ChIP-seq in Proerythroblast from Pinello et al., 201339. ChIP-seq and scRNA-seq data generated in this study are provided in Supplementary Data file 2 and Supplementary Data file 3, respectively. All other data is provided in the Source Data file.

Human research participants

Policy information about studies involving human research participants and Sex and Gender in Research.

Reporting on sex and gender	Nothing to report
Population characteristics	Nothing to report
Recruitment	Nothing to report
Ethics oversight	Nothing to report

Note that full information on the approval of the study protocol must also be provided in the manuscript.

Field-specific reporting

Please select the one below that is the best fit for your research. If you are not sure, read the appropriate sections before making your selection.

☒ Life sciences ☐ Behavioural & social sciences ☐ Ecological, evolutionary & environmental sciences

For a reference copy of the document with all sections, see nature.com/documents/nr-reporting-summary-flat.pdf

Life sciences study design

All studies must disclose on these points even when the disclosure is negative.

Sample size	No sample size was calculated prior to in vivo experiments.
Data exclusions	No data points were excluded.
Replication	Experiments were replicated at least twice unless stated otherwise in the figure legends. All attempts at replication were successful. Technical replicates between experiments were analyzed together.
Randomization	Mice were randomly attributed to test groups in transplantation experiments. In vitro experiments were not randomized.
Blinding	Colony forming assays were scored blindly.

Reporting for specific materials, systems and methods

We require information from authors about some types of materials, experimental systems and methods used in many studies. Here, indicate whether each material, system or method listed is relevant to your study. If you are not sure if a list item applies to your research, read the appropriate section before selecting a response.

Materials & experimental systems

n/a	Involved in the study
	☒ Antibodies
	☒ Eukaryotic cell lines
☒	Palaeontology and archaeology
	☒ Animals and other organisms
☒	Clinical data
☒	Dual use research of concern

Methods

n/a	Involved in the study
	☒ ChIP-seq
	☒ Flow cytometry
☒	MRI-based neuroimaging

Antibodies

Antibodies used: Anti-GATA2 (Abcam, ab173817), Anti-GFI1B (CST, 5849); Anti-FOS (Sigma, SAB4500995); Anti-mCherry (Abcam, ab167453); Anti-Calnexin (Abcam, ab22595); Anti-Histone 3 (Abcam, ab1791); Mouse IgG HRP Linked Whole Ab (GE Healthcare, NXA931); ECL Rabbit IgG, HRP-linked F(ab')2 fragment (GE Healthcare, NA9340); Anti-H3S10p (Abcam, ab47297); Goat Anti-Rabbit IgG H&L, AF488 (Abcam, ab150077), PE/Cy7 anti-human/mouse CD49f (GoH3, BioLegend, 313622); PE anti-human CD9 (HI9a, BioLegend, 312106); PECy5 anti-mouse/human B220 (RA3-6B2, BioLegend, 103210); PECy5 anti-mouse CD3e (145-2C11, BioLegend, 100310); PECy5 anti-mouse/human Mac1 (M1/70, BioLegend, 101210) PECy5 anti-mouse Gr1 (RB6-8C5, BioLegend, 108410); PECy5 anti-mouse Ter119 (TER-119, BioLegend, 116210); FITC anti-mouse CD71 (RI7217, BioLegend, 113806); APC anti-mouse Ter119 (TER-119, BioLegend, 116212); PE anti-mouse Sca-1 (E13-161.7, BioLegend, 122508); APC anti-mouse c-kit (2B8, BioLegend, 105812); PE anti-mouse Ter119 (TER-119, BioLegend, 116208); APCeF780 anti-mouse c-kit (2B8, Invitrogen, 47-1171-82); FITC anti-mouse CD41 (MWReg30,vBD Biosciences, 553848); APC anti-mouse CD16/32 (93, BioLegend, 101326); PE anti-mouse CD45.1 (A20, BioLegend, 110708); FITC anti-mouse CD45.1 (A20, BioLegend, 110706); APC anti-mouse CD45.2 (104, BioLegend, 109814); PE anti-mouse CD45.2 (104, BioLegend, 109808); APC anti-mouse B220 (RA3-6B2, BioLegend, 102312); APC anti-mouse CD3e (145-2C11, BioLegend, 100312); BV421 anti-mouse Sca-1 (D7, BioLegend, 108127); PECy7 anti-mouse CD150 (TC15-12F12.2, BioLegend, 115914); FITC anti-mouse CD48 (HM48-1, BioLegend, 103403) Anti-phospho-Ser/Thr-Pro MPM-2 (Sigma, 05-368); Goat anti-mouse IgG(H+L) AF647 (Invitrogen, A32728); Goat Anti-CD31 (R&D Systems, AF3628); Rabbit Anti-Runx1 (abcam, ab92336); Anti-rabbit AF594 (Invitrogen, A21207); Anti-goat AF647 (Invitrogen, A21447); AF488 anti-rat (Invitrogen, A21208); Anti-human GATA2 (Santa Cruz, sc-9008); Rabbit anti-Cyclin B1 (CST, 4138).

Validation: All antibodies were validated in positive control cell-types.

Eukaryotic cell lines

Policy information about cell lines and Sex and Gender in Research

Cell line source(s): K562 cell line (CCL-243, ATCC) was initially derived from a female with chronic myelogenous leukemia. HEK 293T cell line (CRL-3216, ATCC) was initially obtained from human embryonic kidney carcinoma.

Authentication: None of the cell lines were independently authenticated.

Mycoplasma contamination: Cells were not tested for mycoplasma.

Commonly misidentified lines (See ICLAC register): None.

Animals and other research organisms

Policy information about studies involving animals; ARRIVE guidelines recommended for reporting animal research, and Sex and Gender in Research

Laboratory animals: Only mus musculus were used. 8 to 12-weeks old pregnant C57BL/6J (B6) females were used for mouse embryonic fibroblast isolation; B6.SJL-Ptprca Pepcb/BoyJ (B6.SJL) and C57BL/6JxB6.SJL 11 to 14-weeks old males were used for transplantation assays with MD-Gata2 (129S2;C57BL/6N) derived placenta suspensions; MD-Gata2 (129S2;C57BL/6N) 9 to 13-weeks old males, B6.SJL 11 to 13-weeks old males and C57BL/6JxB6.SJL 9 to 13-weeks old females were used in transplantation assays with LSK-SLAM HSCs. 8 to 12-weeks pregnant MD-Gata2 females were used for hematopoietic embryonic tissue isolation for several experiments.

Wild animals: No wild animals were used.

Reporting on sex: No sex analysis was performed.

Field-collected samples: None.

Ethics oversight: Animal experiments were performed according to the ethical permit protocol 11845/2019 approved by the Malmö - Lund Animal Experimentation Ethics Committee (Malmö - Lunds djurförsöksetiska nämnd).

Note that full information on the approval of the study protocol must also be provided in the manuscript.

ChIP-seq

Data deposition

- [x] Confirm that both raw and final processed data have been deposited in a public database such as GEO.
- [x] Confirm that you have deposited or provided access to graph files (e.g. BED files) for the called peaks.

Data access links<br>*May remain private before publication.*	To access GSE207551 (https://www.ncbi.nlm.nih.gov/geo/query/acc.cgi?acc=GSE207551) use token: olgpuiwonzkndyn
Files in database submission	GSM6294389_Asynchronous-K562-GATA2.bed.gz - Merged called peaks using MACS and Genrich for Asynchronous condition<br>GSM6294390_Mitotic-K562-GATA2.bed.gz - Merged called peaks using MACS and Genrich for Mitotic condition<br>GSM6294389_Asynchronous-K562-GATA2_hg38.bw - bigwig files for GATA2 ChIP-seq in Asynchronous condition<br>GSM6294391_Asynchronous-K562-Input_hg38.bw - bigwig files for Input ChIP-seq in Asynchronous condition<br>GSM6294390_Mitotic-K562-GATA2_hg38.bw - bigwig files for GATA2 ChIP-seq in Mitotic condition<br>GSM6294392_Mitotic-K562-Input_hg38.bw - bigwig files for Input ChIP-seq in Mitotic- condition
Genome browser session<br>(e.g. UCSC)	Use link to access the genome browser session with pre-uploaded tracks http://genome.ucsc.edu/s/ilyak/GATA2-ChipSeq-hg38

Methodology

Replicates	No replicates.
Sequencing depth	Asynchronous-K562-GATA2 - 42875838 total number of reads - 27465796 uniquely mapped reads (mapping quality >= 25) - 75 bp single-end reads<br>Mitotic-K562-GATA2 - 39628110 total number of reads - 21935679 uniquely mapped reads (mapping quality >= 25) - 75 bp single-end reads<br>Asynchronous-K562-Input - 46453751 total number of reads - 37300393 uniquely mapped reads (mapping quality >= 25) - 75 bp single-end reads<br>Mitotic-K562-Input - 48406097 total number of reads - 38526905 uniquely mapped reads (mapping quality >= 25) - 75 bp single-end reads
Antibodies	Anti-human GATA2 (Santa Cruz, sc-9008)
Peak calling parameters	For each file we used current pipeline:<br>bwa aln ref.fa *.fq > *.sai<br>bwa samse ref.fa *.sai *.fq > *.sam<br>samtools markdup -r *.sam - | samtools view -q 25 -b -o *.bam<br><br>Peak calling was done for each condition sseparately<br>macs2 callpeak -t *.bam -c *.bam -f BAM -p 1e-7 2> *.macs2.log<br>Genrich -t *.treat.bam -c *.input.bam -o *.narrowpeak -y -a 175
Data quality	We used MACS cutoff pvalue - 1e-7 and Genrich AUC cutoff - 175. Peak filtering was performed by removing false ChIP-Seq peaks as defined within the ENCODE blacklist. The peaks called from MACS and Genrich were merged together by bedtools merge.<br>Number of peaks in Asynchronous condition - 31777<br>Number of peaks in Mitotic condition - 1648
Software	BWA (v0.7.12); samtools (v1.12); MACS (v2.1.0); Genrich (v0.6.1); deeptools (v2.5.4); bedtools (v2.30); base R (v4.0.3); ChIPpeakAnno R library (v3.24.2); ChIPseeker R library (v1.26.2); Cyclebase 3.0; FIMO (MEME Suite v5.5.0); GREAT v4.0.4.

Flow Cytometry

Plots

Confirm that:

- [ ] The axis labels state the marker and fluorochrome used (e.g. CD4-FITC).
- [x] The axis scales are clearly visible. Include numbers along axes only for bottom left plot of group (a 'group' is an analysis of identical markers).
- [x] All plots are contour plots with outliers or pseudocolor plots.
- [x] A numerical value for number of cells or percentage (with statistics) is provided.

Methodology

Sample preparation	Cells undergoing hemogenic reprogramming were dissociated, pelleted, and incubated with PE-CD9 and PE-Cy7-CD49f antibodies diluted 1:100 in PBS 2% FBS at 4 ºC for 20 min, together with mouse serum 1% (v/v). Single live (DAPI-) cells were analysed. Cells expressing FUCCI vector and mTurquoise fluorescent proteins were analysed directly after collection. Mouse-

	derived blood for donor-contribution analysis was stained with FITC-CD45.1 (1:100), PE-CD45.2 (1:100), APC-B220/CD3 (0.4:100 each), PECy5-B220/Mac1/Gr1 (0.4:100 each), for 20 min on ice. BM isolated from mice 6 months after transplantation were treated with BD Pharm Lyse to remove RBC, washed, filtered, and stained for lineage with PeCy5-Ter119/B220/Gr1/Mac1/CD3e (1:400 each), plus PE-CD45.1 (1:100) and APC-CD45.2 (1:100) antibodies, prior to analysis. Blood cell suspensions from whole embryo bleedings were incubated with lineage antibodies PE-Cy5-B220/Gr1/Mac1/CD3 (1:400 each), 7AAD (dead cell exclusion) and the erythroblast development markers FITC-CD71 and APC-Ter11967 (1:100 each). For EMP analysis, single-cell E9.5 yolk sac suspensions were stained with DAPI, PE-Ter119, APC-eF780-c-kit, FITC-CD41 and APC-CD16/32 (1:100 each), as previously reported68. Nocodazole arrest efficiency of HEK 293T and HDFs was assessed by propidium iodide (PI) staining after fixation with 70% ice-cold ethanol. Prior to analysis, ethanol was washed, and cells resuspended in PI buffer (50 ug/mL PI, 100 ug/mL RNAse A, 0.5% of 10% NP-40) for 20 min on ice and 10 min at room temperature (RT). To check mitotic arrest of K562 for ChIP-seq, asynchronous and nocodazole treated cells were double fixed, stained MPM-2 and resuspended in PI buffer prior to analysis.
Instrument	BD LSR Fortessa, Fortessa X20, LSR II, Aria II and Aria III.
Software	BD FACSDiva and FLOWJO LLC (v10.6.1)
Cell population abundance	K562 fixed cells positive for MPM-2 (mitotic) were FACS sorted, washed and cell pellets were snap-frozen for ChIP. Purity check after sorting confirmed purity of approximately 90%. Mitotic cells were identified in the 4N peak according to propidium iodide (PI) staining.
Gating strategy	For all experiments cell debris were excluded with FSC-A/SSC-A gates and doublets were excluded with FSC-A/FSC-H. Dead cell exclusion (DAPI or 7AAD negative population) was preformed when appropriate. Positive staining was determined based on FMO and single stains for each experiment.

☒ Tick this box to confirm that a figure exemplifying the gating strategy is provided in the Supplementary Information.
